# Effect of natural products use prior to infection with COVID-19 on disease severity and hospitalization: A self-reported cross-sectional survey study

**DOI:** 10.12688/f1000research.121933.1

**Published:** 2022-06-10

**Authors:** Refat M. Nimer, Omar F. Khabour, Samer F. Swedan, Hassan M. Kofahi

**Affiliations:** 1 Department of Medical Laboratory Sciences, Jordan University of Science and Technology, Irbid, 22110, Jordan

**Keywords:** COVID-19, natural products, carnation, onion, lemon, citrus, hospitalization, severity

## Abstract

**Background:** Managing coronavirus disease 2019 (COVID-19) using available resources is essential to reduce the health burden of disease. The severity of COVID-19 is affected by nutritional status. In this study the effect of natural product use prior to infection with COVID-19 on disease severity and hospitalization was explored.

**Methods:** This was a cross-sectional study. Between March and July 2021, a self-administered survey was conducted in Jordan. Individuals who recovered from COVID-19 and were ≥18 years old were the study population. Study measures included the use of natural products, COVID-19 severity, and hospitalization status. A multivariate regression model was used for statistical analysis.

**Results:** The mean age (mean ± SD) of the study sample (n=2,148) was 40.25 ± 15.58 years old. Multivariate logistic regression showed that the regular intake of carnation (OR [0.56], CI [0.37–0.85]), onion (OR [0.69], CI [0.52–0.92]), lemon (OR [0.68], CI [0.51–0.90]), and citrus fruits (OR [0.66], CI [0.50–0.89]) before infection were associated with a substantial reduction in COVID-19 severity (P<0.01). Also, the consumption of carnation (OR [0.55], CI [0.34–0.88]), lemon (OR [0.57], CI [0.42–0.78]), and citrus fruits (OR [0.61], CI [0.44–0.84]) were associated with a significant decrease in the frequency of COVID-19-induced hospitalization (P<0.01).

**Conclusions:** Regular consumption of carnation, lemon, and citrus fruits before infection was associated with better outcomes for COVID-19. Studies on other populations are required to confirm these findings.

## Introduction

Coronavirus disease 2019 (COVID-19) is a global pandemic due to the SARS-CoV-2 that causes severe acute respiratory syndrome. ^1^^,^^2^ Since the World Health Organization (WHO) declared the pandemic in March, 2020, the mortality toll from COVID-19 worldwide has passed six million by April 2022. ^3^ SARS-CoV-2 can cause a wide variety of symptoms, from asymptomatic infection to severe acute respiratory syndrome and death. ^4^^,^^5^

Maintaining a healthy diet has been suggested as a way to boost immune functions and protect against severe infections. ^6^ Certain foods and natural products contain bioactive constituents that have immunomodulating, anti-inflammatory, antioxidant, antibacterial, and antiviral properties. Hence, they can be used for pre- and post-exposure prophylaxis to enhance the activity and quantity of cytokines and different types of white blood cells. ^7^ Therefore, natural products may enhance viral infection outcomes by reducing inflammation and respiratory symptoms. ^8^

Most people in the Middle East region still consume traditional medicinal herbs, immune-boosting foods, and drinks as part of their diet. ^9^^–^^11^ During the pandemic, increased demand on medicinal natural products certain foods, drinks, and medicinal herbs such as ginger, garlic, turmeric, and citrus fruits, has been observed. ^12^^,^^13^

Several studies have examined the effect of consumption of certain foods, drinks, and medicinal herbs on COVID-19 progression. ^14^^,^^15^ However, the method of preparing and consuming these natural products may vary for different populations, which may affect the desired benefits of these products. In addition, each geographical area or country may have unique medicinal herbs and immunomodulatory foods. In Jordan, the effect of regular consumption of certain immune-boosting foods and medicinal herbs prior to infection with COVID-19 on the clinical course has not yet been investigated. Due to the seriousness of COVID-19 infection, we hypothesized that an increase in the use of some immune-boosting foods and medicinal herbs would be associated with a less severe form of COVID-19 infection. Therefore, the current study examined the association between the use of many natural products and foods that boost the immune system (green tea, black caraway, Indian costus, cumin, turmeric, anis, chamomile, propolis, honey, carnation, star anis, onion, garlic, lemon, and citrus fruits) before infection with the risk of COVID-19 severity and hospitalization in Jordan.

## Methods

### Study design and subjects

This cross-sectional survey was carried out in Jordan (between March and July 2021) as part of the Jordanian COVID-19 survey project (JCSP). ^16^ The Institutional Review Board of Jordan University of Science and Technology granted ethical approval for the project (Ref.: 3/139/2021, dated 30/03/2021). Adults who recovered from COVID-19 were included in the current investigation. Exclusion criteria included current COVID-19 infection and vaccination prior to contracting the virus. Persons previously vaccinated were excluded since vaccination has a considerable influence on disease severity and may mask the potential benefits of the consumption of medicinal herbs and immune-boosting foods on the dependent variables of the study. This cross-sectional study was designed as recommended by the STROBE checklist. ^17^

### Study instrument

The current study used a self-administered questionnaire in Arabic, which can be found translated into English as *Underlying data.* ^64^ Participants were asked about their demographics (age, gender, body mass index (BMI), and education), comorbidities, and consumption of medicinal herbs and immune-boosting foods before infection with COVID-19. Furthermore, infection symptoms and admission to hospital data were gathered. A panel of area specialists validated the questionnaire, which was then piloted on a limited number of participants. The opinions of experts and participants were gathered and utilized to assess and enhance the clarity of the questionnaire. Results of the first draft of the questionnaire were excluded from the final analysis. The principal investigators’ involvement in data collection was minimized to reduce sources of bias. Participants were chosen to represent various Jordanian geographical regions, and information was gathered from them through an online questionnaire supplied to participants by trained research assistants. We used specific techniques to reduce response bias as much as possible. First, the researcher did not interpret the questionnaire because it was self-administered. Second, we double-checked the items’ clarity by translating them into Arabic. Finally, all duplicate entries were eliminated.

### Sample size calculation

The sample size for our study was calculated using the online Raosoft sample size calculator (Raosoft Inc., Seattle, WA, USA). According to the world meter elaboration of the most recent United Nations data, Jordan’s population is around 10 million people. The confidence level was set at 95%, the margin of error was set at 3%, and the response distribution was set at 50%. With 770,712 confirmed cases reported by the end of July 2021, ^18^ the recommended sample size was 1067.

### Recruitment of subjects

A convenient sampling procedure was used in the study. “Google Forms” was used to create and administer the survey. To ensure anonymity, the study did not collect identifying information, such as participants’ names and places of work. In addition, the data were saved in encrypted digital format that required a password to access. The first section of the questionnaire sought informed consent and confirmed recovery from COVID-19. All paricipants provided informed consent. Anyone could opt out and withdraw from the survey by not submitting their answers, so participation was entirely voluntary. To ensure that the Jordanian population is adequately represented, trained researchers recruited participants from various Jordanian governorates. The questionnaire was completed by a total of 2,148 participants.

### Classification of COVID-19 severity

Based on symptoms, the severity of COVID-19 infection was classified into two categories; severe and non-severe (asymptomatic, mild symptoms, and moderate), as previously reported. ^19^ Fever, sore throat, body pains, and nausea, with no signs or symptoms of pneumonia, were considered mild cases. Pneumonia (persistent fever and cough) but no hypoxemia (arterial oxygen saturation measured by pulse oximetry (SpO _2_) ≤ 92%) were considered moderate cases. ^19^ Confirmed severe pneumonia and hypoxemia were considered severe cases. It is worth noting that during peak COVID-19 waves, several patients were not admitted to hospitals because of the increased burden on hospitals. ^16^ As a result, such cases received medical care at home through private doctor visits, and some used medical oxygen supply systems in their homes.

### Statistical analysis

In this study, the independent variables were the use of natural products (medicinal herbs, immune-boosting foods), whereas the dependent variables were COVID-19 severity and hospitalization. All data with missing values were excluded from the analysis. For different variables, percentages, frequencies, means, and standard deviations (SD), were used as appropriate. The Chi-squared test was utilized to determine any differences in severity/hospitalization between users of natural products. Multivariate logistic regression was applied to examine the impact of natural food use on the severity/hospitalization while controlling for different confounders. The adjusted odds ratios (OR) and 95% confidence interval (CI) were presented. P<0.05 indicates statistical significance. The data were analyzed using IBM SPSS Statistics v23 (RRID:SCR_016479).

## Results

### Demographic characteristics of the participants

The questionnaire was completed by 2,148 participants. The demographics of the sample are summarized in Table 1. ^64^ Women comprised 58.2% of participants. The participants’ mean age was 40.25±15.58 years. The majority of participants had non-severe COVID-19 infection and were not admitted to hospitals (87.9% and 89.8%, respectively). Among the participants, 16.9% were smokers, and 23.1% had at least one comorbidity ( Table 1).

**Table 1.** Demographics of the sample.

Characteristics	Value (n=2,148)
Reported age (years) *	40.25±15.58
Male: Female ratio	0.72: 1.00
Body mass index *	28.17±7.38
Tobacco use ^#^	362 (16.85)
Chronic diseases ^#^	496 (23.09)
COVID-19 infection status	
Severe disease ^#^	260 (12.10)
Non-severe disease ^#^	1,888 (87.89)
Admitted to hospital ^#^	219 (10.19)

* Expressed as: mean ± SD.

^#^ Expressed as N (%). COVID-19, coronavirus disease 2019.

### Association of natural products use with COVID-19 severity and hospitalization

COVID-19 severity/hospitalizations were investigated in relation to the use of medicinal herbs and immune-boosting foods ( Table 2). Lemon and citrus fruits were associated with lower incidence of severe COVID-19 and hospitalization (P<0.01). Moreover, intake of ginger and green tea was associated with reduced disease severity (P=0.018 and P=0.036, respectively). However, no significant effects on severe COVID-19 and hospitalization outcomes were observed due to consumption of anise, chamomile, propolis, honey, onions, garlic, carnation, star anise, black caraway, Indian costus, and turmeric, prior to infection.

**Table 2.** Severe COVID-19 and hospitalization cases based on natural product use prior to infection.

		COVID-19 classification					Hospitalized				
		Non-severe		Severe		P-value	No		Yes		P-value
		n	%	n	%		n	%	n	%	
Did you consume anise before COVID infection to boost your immune system	No	1,029	87.4%	148	12.6%	0.43	1,053	89.5%	124	10.5%	0.40
	Yes	827	88.5%	107	11.5%		846	90.6%	88	9.4%	
Did you consume chamomile before COVID infection to boost your immune system	No	1,229	87.7%	173	12.3%	0.44	1,263	90.1%	139	9.9%	0.85
	Yes	628	88.8%	79	11.2%		635	89.8%	72	10.2%	
Did you consume propolis before COVID infection to boost your immune system	No	1,714	87.9%	235	12.1%	0.78	1,755	90.0%	194	10.0%	0.30
	Yes	101	87.1%	15	12.9%		101	87.1%	15	12.9%	
Did you consume honey before COVID infection to boost your immune system	No	1,023	88.3%	135	11.7%	0.69	1,050	90.7%	108	9.3%	0.27
	Yes	840	87.8%	117	12.2%		854	89.2%	103	10.8%	
Did you consume onions before COVID infection to boost your immune system	No	993	87.1%	147	12.9%	0.21	1,023	89.7%	117	10.3%	0.78
	Yes	863	88.9%	108	11.1%		875	90.1%	96	9.9%	
Did you consume garlic before COVID infection to boost your immune system	No	1,075	88.2%	144	11.8%	0.78	1,100	90.2%	119	9.8%	0.57
	Yes	784	87.8%	109	12.2%		799	89.5%	94	10.5%	
Did you consume carnation before COVID infection to boost your immune system	No	1,515	87.5%	217	12.5%	0.12	1,552	89.6%	180	10.4%	0.21
	Yes	320	90.4%	34	9.6%		325	91.8%	29	8.2%	
Did you consume star anise before COVID infection to boost your immune system	No	1,580	87.6%	224	12.4%	0.20	1,619	89.7%	185	10.3%	0.40
	Yes	251	90.3%	27	9.7%		254	91.4%	24	8.6%	
Did you consume ginger before COVID infection to boost your immune system	No	1,157	86.9%	175	13.1%	**0.02** *	1,189	89.3%	143	10.7%	0.10
	Yes	708	90.3%	76	9.7%		717	91.5%	67	8.5%	
Did you consume green tea before COVID infection to boost your immune system	No	1450	87.3%	211	12.7%	**0.04** *	1,486	89.5%	175	10.5%	0.08
	Yes	393	91.0%	39	9.0%		399	92.4%	33	7.6%	
Did you consume lemons before COVID infection to boost your immune system	No	674	86.1%	109	13.9%	**0.02** *	687	87.7%	96	12.3%	**0.003** *
	Yes	1,194	89.6%	139	10.4%		1,223	91.7%	110	8.3%	
Did you consume citrus fruits before COVID infection to boost your immune system	No	574	84.8%	103	15.2%	**0.001** *	587	86.7%	90	13.3%	**<0.001** *
	Yes	1,299	89.7%	149	10.3%		1,327	91.6%	121	8.4%	
Did you consume black caraway before COVID infection to boost your immune system	No	1,503	87.8%	208	12.2%	0.44	1,537	89.8%	174	10.2%	0.65
	Yes	333	89.3%	40	10.7%		338	90.6%	35	9.4%	
Did you consume Indian costus before COVID infection to boost your immune system	No	1,736	87.9%	238	12.1%	0.56	1,773	89.8%	201	10.2%	0.53
	Yes	97	89.8%	11	10.2%		99	91.7%	9	8.3%	
Did you consume cumin before COVID infection to boost your immune system	No	1,502	88.1%	202	11.9%	0.70	1,533	90.0%	171	10.0%	0.98
	Yes	341	87.4%	49	12.6%		351	90.0%	39	10.0%	
Did you consume turmeric before COVID infection to boost your immune system	No	1,554	88.0%	211	12.0%	0.91	1,586	89.9%	179	10.1%	0.46
	Yes	289	87.8%	40	12.2%		300	91.2%	29	8.8%	

COVID-19, coronavirus disease 2019.

* P≤0.05.

### Regression model

The multivariate logistic regression model was performed to account for potential confounders and comorbidities. The data are shown in Table 3 for two outcomes: COVID-19 severity and hospitalization status with the use of each natural product. After controlling for covariates, the findings ( Table 3) showed that consumption of carnation (P<0.01), onion (P<0.01), lemon (P<0.01), and citrus fruits (P<0.01) were predictors of lower disease severity. In addition, the consumption of carnation (P<0.01), lemon (P<0.001), and citrus fruits (P<0.01), were predictors of reduced hospitalizations due to COVID-19 infection ( Table 3).

**Table 3.** Logistic regression analyses for medicinal herbals, natural products, and immune-boosting foods used with COVID-19 severity and hospitalization as the output.

Substance consumed before COVID-19	Total number of users	Severity			Hospitalization		
		P-value	Odds ratio (OR)	95% CI of OR	P-value	Odds ratio (OR)	95% CI of OR
Green Tea	432	0.13	0.75	0.51–1.10	0.26	0.78	0.52–1.19
Black caraway	373	0.07	0.70	0.48–1.04	0.11	0.71	0.47–1.08
Indian Costus	108	0.09	0.55	0.27–1.09	0.08	0.50	0.24–1.08
Cumin	390	0.90	0.98	0.68–1.40	0.56	0.89	0.60–1.33
Turmeric	329	0.47	0.86	0.58–1.28	0.08	0.67	0.42–1.04
Anis	934	0.32	0.86	0.65–1.15	0.29	0.84	0.62–1.16
Chamomile	707	0.11	0.78	0.57–1.06	0.54	0.90	0.65–1.25
Propolis	116	0.54	0.83	0.45–1.51	0.99	1.00	0.54–1.85
Honey	957	0.33	0.87	0.65–1.16	0.78	0.96	0.70–1.31
Carnation	354	0.01 *	0.56	0.37–0.85	0.01 *	0.55	0.35–0.87
Star Anis	278	0.06	0.65	0.41–1.03	0.15	0.70	0.43–1.13
Onion	971	0.01 *	0.69	0.52–0.92	0.07	0.75	0.55–1.02
Garlic	893	0.25	0.84	0.63–1.13	0.31	0.85	0.62–1.16
Lemon	1333	0.01 *	0.68	0.51–0.90	<0.001 *	0.57	0.42–0.78
Citrus fruits	1448	0.01 *	0.66	0.50–0.89	0.002 *	0.61	0.44–0.84

Each independent variable was adjusted for confounders, and comorbidities.

COVID-19, coronavirus disease 2019.

* P≤0.05.

## Discussion

COVID-19 can show a spectrum of clinical symptoms from mild to severe respiratory distress and death. It is suggested that the nutritional status of people may influence COVID-19 clinical severity. ^20^ In the current study, the effects of using natural products prior to infection with COVID-19 on disease severity and hospitalization were examined. Previous studies have examined the associations between the severity of infection with the intake of immune-boosting foods and medicinal herbs. ^12^^,^^21^^,^^22^ According to the literature, there is no evidence that natural products and medicinal herbs impart protection from or cure COVID-19. ^23^ However, consumption of natural products before becoming infected with COVID-19 may boost the immune system and lead to favored disease outcomes. The current study found that regular consumption of carnation, lemon, and citrus fruits, before infection was associated with better outcomes for COVID-19.

This study found that carnation use by the participants reduced COVID-19 hospitalizations and disease severity. Due to its therapeutic uses, carnation is nominated as a candidate for the management of COVID-19. ^24^^–^^27^ During the COVID-19 pandemic, the consumption of carnation to relieve throat pain has increased. ^28^ A randomized clinical trial of a blend that contains carnation buds demonstrated a boost in energy levels among post-COVID-19 female patients. ^29^ In addition, carnation has antibacterial, antiviral, and antifungal effects, and is used to kill germs in dental creams, toothpaste, mouthwash formulations, and throat sprays. ^22^^,^^30^ The mechanism by which carnation may reduce the severe form of COVID-19 involves interference of SARS-CoV-2 S1-protein binding to the angiotensin-converting enzyme 2 (ACE2) receptor. ^31^ Moreover, carnation has been shown to increase the availability of oxygen by improving blood supply to both the heart and the brain. ^32^ Carnation has also been reported to be beneficial for the management of chronic coughs, shortness of breath, and maintaining a normal heart rate. ^33^

The current study also showed a significant decrease in the frequency of the severe form of COVID-19 and hospitalization due to the consumption of lemon and citrus. Citrus fruits are known to contain a variety of bioactive constituents, including vitamin C, anthocyanin, and flavanones, ^34^ which offer a variety of health benefits, such as the normalization of oxidants and inflammation. ^35^^,^^36^ Furthermore, citrus fruits, such as oranges and grapefruits, are grown in Jordan, making them a readily available natural source of vitamin C. ^37^ Citrus fruits are commonly used by Jordanians to improve the body’s immunity against COVID-19 infection. ^11^ Similar to carnation, active ingredients in citrus fruits can interfere with binding of SARS-CoV-2 to the ACE2 receptor. ^38^^,^^39^ Moreover, naringin of citrus fruits has been shown to suppress the expression of proinflammatory cytokines during the inflammatory response. ^38^^,^^40^ Furthermore, hesperidin, a biologically active molecule in orange fruit, was shown to inactivate the SARS-CoV-2 RNA polymerase complex. ^41^^,^^42^ The antioxidant components of citrus fruits can also protect against oxidative stress associated with COVID-19 infection. ^43^

Onion has been shown to significantly reduce the frequency of severe illness among participants who consumed it regularly. Onion has antiviral, antifibrotic, antioxidant, and anti-inflammatory bioactive compounds, such as quercetin, apigenin, and selenium. ^36^^,^^44^ In several studies, onion and its bioactive components reduced inflammation in the lungs and protected against various respiratory illnesses. ^45^^–^^47^ It is suggested that phytochemicals derived from onions can interfere with the function of the main protease of SARS-CoV-2. ^48^^–^^50^ In addition, anti-inflammatory diets that incorporate onions were recommended to reduce the severity of COVID-19. ^12^^,^^51^^,^^52^

Although honey and ginger were among the most consumed natural products for the prevention or mitigation of COVID-19 symptoms in Jordan, ^11^ results showed no impact of ginger and honey on the rate of hospitalization and disease severity. Ginger is suggested as a natural product for the management of COVID-19. ^53^ In contrast to the current findings, Aldwihi *et al*., showed that patients who consumed ginger were less likely to be hospitalized. ^54^ Moreover, a decrease in COVID-19 severity was observed among patients who consumed ginger as treatment. ^55^ According to clinical trials, honey (propolis), a resinous substance made by bees, may help reduce viral clearance time and improve clinical COVID-19 outcomes by interfering with viral replication and entry. ^56^^–^^58^ However, the effects of honey were observed in a limited number of studies involving small sample sizes. ^59^ Thus, more investigations are required to confirm the role of bee products in the management of COVID-19.

According to the logistic regression analysis, no associations were observed due to consumption of green tea, black caraway, Indian costus, cumin, turmeric, anis, chamomile, star anis, and garlic with COVID-19 hospitalizations and severity. While several studies have revealed the anti-inflammatory and antiviral effects of many of these natural products, ^60^^–^^62^ their contribution toward reducing COVID-19 severity is highly controversial, and requires preclinical and clinical trial evaluations, and validation using models of the disease. ^8^^,^^63^

Among the study limitations is that the total number and quantity of natural products used by the subjects were unknown. Furthermore, data on the duration of COVID-19 and hospitalization were not collected. Finally, since the study collected retrospective data, the data may be subject to recall bias. As a result, more studies are required to verify the study findings.

## Conclusions

The study findings showed that the regular consumption of lemon, citrus fruits, and carnation lowers the rates of COVID-19 severity and hospitalization. Studies in other populations are required to confirm these findings.

## Data availability

### Underlying data

Figshare: Natural Products Raw Data. https://doi.org/10.6084/m9.figshare.19758820. ^64^

This project contains the following underlying data:

- Natural products Raw Data.xlsx (survey responses from all participants)
- Questionnaire in English.docx

Data are available under the terms of the Creative Commons Attribution 4.0 International license (CC-BY 4.0).
