# Supplementary material for: Effect of natural products use prior to infection with COVID-19 on disease severity and hospitalization: A self-reported cross-sectional survey study
Source: F1000Res. 2022 Jul 11;11:639. Originally published 2022 Jun 10. [Version 2] doi: 10.12688/f1000research.121933.2 (PMC9294495; doi:10.12688/f1000research.121933.2)
Supplement: Peer review history

# Peer review: Reviewer response for version 2

**DOI:** 10.5256/f1000research.135778.r143818

**Published:** 2022-07-18

**Authors:** Caterina Vicidomini

**Affiliations:** 1 Istituto di Biostrutture e Bioimmagini IBB-CNR, Naples, Italy

The changes made are satisfactory. I have no further comments to add. The paper is approved.

Is the work clearly and accurately presented and does it cite the current literature?

Partly

If applicable, is the statistical analysis and its interpretation appropriate?

Partly

Are all the source data underlying the results available to ensure full reproducibility?

Yes

Is the study design appropriate and is the work technically sound?

Yes

Are the conclusions drawn adequately supported by the results?

Yes

Are sufficient details of methods and analysis provided to allow replication by others?

Yes

Reviewer Expertise:

Environmental chemistry & human health. Computational studies. Synthesis of small molecules

I confirm that I have read this submission and believe that I have an appropriate level of expertise to confirm that it is of an acceptable scientific standard.

# Peer review: Reviewer response for version 2

**DOI:** 10.5256/f1000research.135778.r143817

**Published:** 2022-07-13

**Authors:** Fatin Y. Atrooz

**Affiliations:** 1 Department of Pharmacological and Pharmaceutical Sciences, College of Pharmacy, University of Houston, Houston, TX, USA

Approved.

Is the work clearly and accurately presented and does it cite the current literature?

Yes

If applicable, is the statistical analysis and its interpretation appropriate?

Yes

Are all the source data underlying the results available to ensure full reproducibility?

Yes

Is the study design appropriate and is the work technically sound?

Partly

Are the conclusions drawn adequately supported by the results?

Yes

Are sufficient details of methods and analysis provided to allow replication by others?

Partly

Reviewer Expertise:

Behavioral neuroscience, cytogenetics, public health research.

I confirm that I have read this submission and believe that I have an appropriate level of expertise to confirm that it is of an acceptable scientific standard.

# Peer review: Reviewer response for version 1

**DOI:** 10.5256/f1000research.133852.r140506

**Published:** 2022-06-28

**Authors:** Caterina Vicidomini

**Affiliations:** 1 Istituto di Biostrutture e Bioimmagini IBB-CNR, Naples, Italy

In this paper, the authors retrospectively explored the effect of a series of natural products use prior to infection with COVID-19 on disease severity and hospitalization. The authors hypothesized that an increase in the use of some immune-boosting foods and medicinal herbs would be associated with a less severe form of COVID-19 infection. Study measures included the use of selected natural products, COVID-19 severity, and hospitalization status. A multivariate regression model was used for statistical analysis.

 In my opinion, this paper has some limitations including those the authors have described in the text. The total number and quantity of natural products used by the subjects were unknown, and also, I didn't find any information on whether the participants consumed these natural products as commercial supplements, or if they prepared them from natural sources at home, for example.

 In any case, the work seems interesting and provides ideas for research insights. In my opinion it is therefore worthy of indexing.

 I have just a few suggestions:

- Provide some information on the recruitment method adopted.
- Specify, at least for the most active products such as carnation, how it is usually consumed.
- Reading the questionnaire, I saw that the authors collected data on potential confounders and comorbidities, but they didn't specify anything on this aspect. Please, add what they considered confounders. If possible, it would be interesting to evaluate whether, for example, the cigarette smoking modified the beneficial effect of natural substances intake.
- I know that there are other natural substances traditionally used to boost the immune system, but they were not mentioned or considered in the study. Please add a comment in the text and cite at least the following reference. ^1^

Is the work clearly and accurately presented and does it cite the current literature?

Partly

If applicable, is the statistical analysis and its interpretation appropriate?

Partly

Are all the source data underlying the results available to ensure full reproducibility?

Yes

Is the study design appropriate and is the work technically sound?

Yes

Are the conclusions drawn adequately supported by the results?

Yes

Are sufficient details of methods and analysis provided to allow replication by others?

Yes

Reviewer Expertise:

Environmental chemistry & human health. Computational studies. Synthesis of small molecules

I confirm that I have read this submission and believe that I have an appropriate level of expertise to confirm that it is of an acceptable scientific standard, however I have significant reservations, as outlined above.

## References

[1] : Spices and herbs: Potential antiviral preventives and immunity boosters during COVID-19. *Phytother Res* .2021; 10.1002/ptr.7019 10.1002/ptr.7019 33511704PMC8013177

# Response

**Published:** 2022-07-02

**Authors:** Refat Nimer

**Affiliations:** Jordan University of Science and Technology, Jordan

We would like to thank the reviewers for careful and thorough reading of this manuscript and for the thoughtful comments and constructive suggestions, which helped to improve the quality of this manuscript.

 Please find attached a point-by-point response to reviewers’ concerns. We hope that you find our responses satisfactory and that the manuscript is now acceptable for publication.

Reviewer #2 (Comments to the Author): 

 In my opinion, this paper has some limitations including those the authors have described in the text. The total number and quantity of natural products used by the subjects were unknown,..

**Authors' response:** We agree with the reviewer that the rate of consumption of these natural products was unknown. However, this point was mentioned in the limitations (highlighted in red).

 And also, I didn’t find any information on whether the participants consumed these natural products as commercial supplements, or if they prepared them from natural sources at home, for example.

**Authors’ response:** This study focuses on natural products, not commercial supplements.  However, the point raised by the reviewer is now incorporated in the revised manuscript.

 Provide some information on the recruitment method adopted.

**Authors' response:** More information was added at “Study design and subjects” section.

 Specify, at least for the most active products such as carnation, how it is usually consumed.

**Authors’ response:** Your suggestion is now incorporated in the updated version of the manuscript.

 Reading the questionnaire, I saw that the authors collected data on potential confounders and comorbidities, but they didn't specify anything on this aspect. Please, add what they considered confounders. If possible, it would be interesting to evaluate whether, for example, the cigarette smoking modified the beneficial effect of natural substances intake.

**Authors’ response:** COVID-19 was reported to be more severe in people having high BMI or comorbidities. However, the aim of this study was to explore the effect of using natural products on the severity of the disease, regardless of the confounding factors. Therefore, multivariate regression analysis was applied to control for possible confounding factors such as age, gender, cigarette smoking, and the number of comorbidities (Table 3). The analysis without adjusted confounding factors is shown in Table 2.

 The point raised by the reviewer is now discussed in the revised manuscript. 

 I know that there are other natural substances traditionally used to boost the immune system, but they were not mentioned or considered in the study. Please add a comment in the text and cite at least the following reference. PMID: 33511704

**Authors’ response: **We agree with the reviewer's comment. However, this study covered the broadest range of natural products used by the Jordanian population.

 The point raised by the reviewer is now incorporated in the revised manuscript the last sentence in the introduction) and the proposed reference was added (ref no. 14).

# Peer review: Reviewer response for version 1

**DOI:** 10.5256/f1000research.133852.r140507

**Published:** 2022-06-22

**Authors:** Fatin Y. Atrooz

**Affiliations:** 1 Department of Pharmacological and Pharmaceutical Sciences, College of Pharmacy, University of Houston, Houston, TX, USA

**Summary**: The study explored the association between using specific natural products prior to COVID-19 infection and the severity of the disease and hospitalization status in a sample of Jordanian adults.

**Title**: This is a retrospective study, the study design included a survey about prior COVID-19 infection and natural products consumption prior to infection.

**Methods**:

 - Recruitment method is not clear, how the investigators reached out to the potential participants is not stated. The study suggests that only people who were previously infected with COVID-19 were included in the study. This assumption should be discussed in the inclusion criteria.

 - Calculation of the study sample assumed that the total population is the number of COVID-19 infected people as reported by the end of July 2021. Does this report include only adults or total confirmed cases? As the survey targeted only adults, the authors should identify precisely the study population as a reference for sample size calculation.

**Results**: 

 - Why is lemon considered a separate category from citrus? I suggest including all citrus fruit, including lemon as one category.

 - In table 1, the results were expressed in a different format, I suggest having all the results expressed as N (%). For this purpose, age can be categorized into 2-3 categories, and same for BMI.

 - In the multivariate logistic regression, the authors mentioned that after controlling for covariate, the findings...

 What are the covariates? The authors should list the covariates here, and if that includes controlling for age and gender.

Is the work clearly and accurately presented and does it cite the current literature?

Yes

If applicable, is the statistical analysis and its interpretation appropriate?

Yes

Are all the source data underlying the results available to ensure full reproducibility?

Yes

Is the study design appropriate and is the work technically sound?

Partly

Are the conclusions drawn adequately supported by the results?

Yes

Are sufficient details of methods and analysis provided to allow replication by others?

Partly

Reviewer Expertise:

Behavioral neuroscience, cytogenetics, public health research.

I confirm that I have read this submission and believe that I have an appropriate level of expertise to confirm that it is of an acceptable scientific standard, however I have significant reservations, as outlined above.

# Response

**Published:** 2022-07-02

**Authors:** Refat Nimer

**Affiliations:** Jordan University of Science and Technology, Jordan

We would like to thank the reviewers for careful and thorough reading of this manuscript and for the thoughtful comments and constructive suggestions, which helped to improve the quality of this manuscript.

 Please find attached a point-by-point response to reviewers’ concerns. We hope that you find our responses satisfactory and that the manuscript is now acceptable for publication.

Reviewer #1 (Comments to the Author): 

 - Recruitment method is not clear, how the investigators reached out to the potential participants is not stated. The study suggests that only people who were previously infected with COVID-19 were included in the study. This assumption should be discussed in the inclusion criteria.

**Authors’ response:** The recruitment method has now been illustrated in the revised manuscript. In the cover page for the questionnaire, we outlined the criteria for participation in the study, which includes infection with COVID-19 and recovery from illness. The research assistance emphasized that participants' infection should have been confirmed by polymerase chain reaction (PCR). Participants were approached in public places such as bus stations, parks, religious places, universities and restaurants.

 - Calculation of the study sample assumed that the total population is the number of COVID-19 infected people as reported by the end of July 2021. Does this report include only adults or total confirmed cases? As the survey targeted only adults, the authors should identify precisely the study population as a reference for sample size calculation.

**Authors' response:** The sample size for our study was calculated using Raosoft Online Sample Size Calculator (Raosoft Inc., Seattle, WA, USA). 770,712 is the total confirmed cases reported by the end of July 2021, and the recommended sample size was 1067. However, this calculation is to check whether the sample size in our study is sufficient and representative. The number of cases under 18 years of age during the time of sample collection (before the emergence of the Omicron variant) was expected to be small compared to the adult cases (no data available). Therefore, if patients under 18 years are subtracted from the total cases, this would lead to a negligible change in the sample size. Nonetheless, the study sample size was larger than required (n= 2,148) even if child cases are considered. This point is now clarified in the Method section.

 - Why is lemon considered a separate category from citrus? I suggest including all citrus fruit, including lemon as one category.

**Authors’ response:  **Thank you for your comment. We prefer to keep lemon as a separate category from citrus because some studies showed variations in their nutritional values. This was clarified in the revised manuscript.

 - In table 1, the results were expressed in a different format, I suggest having all the results expressed as N (%). For this purpose, age can be categorized into 2-3 categories, and same for BMI.

**Authors' response: **Done as suggested.

 - In the multivariate logistic regression, the authors mentioned that after controlling for covariate, the findings...

 What are the covariates? The authors should list the covariates here, and if that includes controlling for age and gender.

**Authors' response:** The point raised by the reviewer is now incorporated in the revised manuscript.
